# Prognostic impact of left ventricular diastolic function in patients with septic shock

**DOI:** 10.1186/s13613-016-0136-6

**Published:** 2016-04-21

**Authors:** Céline Gonzalez, Emmanuelle Begot, François Dalmay, Nicolas Pichon, Bruno François, Anne-Laure Fedou, Catherine Chapellas, Antoine Galy, Claire Mancia, Thomas Daix, Philippe Vignon

**Affiliations:** Medical-Surgical ICU, Dupuytren Teaching Hospital, Limoges, France; Inserm CIC 1435, Dupuytren Teaching Hospital, Limoges, France; Department of Biostatistics, Dupuytren Teaching Hospital, Limoges, France; UMR 1092, University of Limoges, Limoges, France; Réanimation Polyvalente, CHU Dupuytren, 2 Ave. Martin Luther King, 87000 Limoges, France

**Keywords:** Septic shock, Cardiac dysfunction, Diastolic dysfunction, Echocardiography, Mortality, Prognosis

## Abstract

**Background:**

Left ventricular (LV) diastolic dysfunction is highly prevalent in the general population and associated with a significant morbidity and mortality. Its prognostic role in patients sustaining septic shock in the intensive care unit (ICU) remains controversial. Accordingly, we investigated whether LV diastolic function was independently associated with ICU mortality in a cohort of septic shock patients assessed using critical care echocardiography.

**Methods:**

Over a 5-year period, patients hospitalized in a Medical–Surgical ICU who underwent an echocardiographic assessment with digitally stored images during the initial management of a septic shock were included in this retrospective single-center study. Off-line echocardiographic measurements were independently performed by an expert in critical care echocardiography who was unaware of patients’ outcome. LV diastolic dysfunction was defined by the presence of a lateral *E*′ maximal velocity <10 cm/s. A multivariate analysis was performed to determine independent risk factors associated with ICU mortality.

**Results:**

Among the 540 patients hospitalized in the ICU with septic shock during the study period, 223 were studied (140 men [63 %]; age 64 ± 13 years; SAPS II 55 ± 18; SOFA 10 ± 3; Charlson 3.5 ± 2.5) and 204 of them (91 %) were mechanically ventilated. ICU mortality was 35 %. LV diastolic dysfunction was observed in 31 % of patients. The proportion of LV diastolic dysfunction tended to be higher in non-survivors than in their counterparts (28/78 [36 %] vs. 41/145 [28 %]: *p* = 0.15). Inappropriate initial antibiotic therapy (OR 4.17 [CI 95 % 1.33–12.5]: *p* = 0.03), maximal dose of vasopressors (OR 1.38 [CI 95 % 1.16–1.63]: *p* = 0.01), SOFA score (OR 1.16 [CI 95 % 1.02–1.32]: *p* = 0.02) and lateral *E*′ maximal velocity (OR 1.12 [CI 95 % 1.01–1.24]: *p* = 0.02) were independently associated with ICU mortality. After adjusting for the SAPS II score, inappropriate initial antibiotic therapy and maximal dose of vasopressors remained independent factors for ICU mortality, whereas a trend was only observed for lateral *E*′ maximal velocity (OR 1.11 [CI 95 % 0.99–1.23]: *p* = 0.07).

**Conclusion:**

The present study suggests that LV diastolic function might be associated with ICU mortality in patients with septic shock. A multicenter prospective study assessing a large cohort of patients using serial echocardiographic examinations remains required to confirm the prognostic value of LV diastolic dysfunction in septic shock.

## Background

Septic shock remains the leading cause of death in the intensive care unit (ICU), with an increasing incidence and a current mortality of approximately 30 % [1–3]. Many experimental and clinical studies extensively documented the presence of a reversible sepsis-induced cardiomyopathy which involves the systolic and diastolic functions of both ventricles [4–7]. The pathophysiology of septic cardiomyopathy is complex [8], and its prognostic impact remains controversial [9–11].

Heart failure with preserved ejection fraction is highly prevalent in the general population and associated with a high morbidity and mortality [12, 13]. In ICU patients, left ventricular (LV) diastolic dysfunction is frequently depicted using Doppler echocardiography [14]. Specifically, reversible abnormal LV relaxation has been reported during the early phase of septic shock [15, 16]. A recent meta-analysis suggests that LV diastolic dysfunction is associated with the mortality of severe sepsis and septic shock [17]. However, this meta-analysis suffered from substantial limitations. First, the size of study populations varied greatly, leading to a wide range of prevalence of LV diastolic dysfunction (20–57 %). Second, two cohorts involved two-third of the global population included in the meta-analysis and were enrolled by the same investigating center [9, 18]. Third, the studied population was heterogeneous since patients were hemodynamically assessed for either a severe sepsis or a septic shock [17]. Accordingly, we sought to assess the potential impact of LV diastolic function on ICU survival in a population of septic shock patients, with respect to other known prognostic factors.

## Methods

This descriptive, retrospective, single-center study was approved by our institutional ethics committee which waived the need for informed consent.

### Study population

Patients were eligible if (1) they were hospitalized in the Medical–Surgical ICU of Limoges University Hospital between December 2009 and December 2014 with a septic shock; (2) they were hemodynamically assessed using transthoracic (TTE) or transesophageal echocardiography (TEE) within the first 24 h of shock, and (3) digital recording of the echocardiographic examination was available for off-line measurements. Patients’ characteristics on ICU admission, comorbidities assessed by the Charlson’s score [19] and severity scores including the Simplified Acute Physiology Score (SAPS) II score [20] and the Sequential Organ Failure Assessment (SOFA) score [21] were recorded. In each patient, the following information was retrieved from electronic medical records or paper charts, as required: site of infection, time lag between the development of septic shock and the first administration of antibiotic therapy, appropriateness of initial antibiotic therapy and volume of fluids administered prior to hemodynamic assessment. During the ICU stay, the following parameters were recorded: maximal dose of catecholamines, use of substitutive steroids therapy, invasive mechanical ventilation and renal replacement therapy. At the time of septic shock diagnosis, the following biological values were obtained: pH, PaO_2_/FiO_2_ ratio, lactate, platelet count, serum creatinine, bilirubin, alanine aminotransferase and the presence of a disseminated intravascular coagulation [22]. Dobutamine was administered in the presence of severe LV systolic dysfunction without fluid responsiveness but persisting signs of tissue hypoperfusion, and no patient received beta-blockers, milrinone or levosimendan.

### Definitions

Sepsis was defined by the presence of at least two criteria of systemic inflammatory response syndrome associated with a clinically or microbiologically documented, or a highly suspected infection. Severe sepsis was defined as a sepsis associated with at least one organ failure different from that responsible for the infection. Septic shock was defined as a severe sepsis associated with low blood pressure despite adequate vascular filling which required a vasopressor support [23, 24]. The onset of septic shock was defined by the time of the first occurrence of recurrent or persistent hypotension (systolic blood pressure <90 mmHg or mean blood pressure <65 mmHg) despite adequate fluid loading (i.e., at least 30 mL/kg) [24]. Appropriateness of initial antibiotic therapy referred to the susceptibility of isolated microorganism rather than the time lag prior to its initiation. Previously determined rules to evaluate appropriateness of initial antibiotic therapy have been followed [25, 26]. Antibiotic therapy was considered appropriate if the first-line treatment included at least one antibiotic active in vitro on the isolated microorganism. When no microorganism was found, the antibiotic therapy was considered appropriate if it was in accordance with broadly accepted guidelines for the expected site infection [25]. This information was recorded by an independent investigator who was unaware of patients’ outcome.

### Echocardiography

Hemodynamic assessment was performed by experienced intensivists highly trained in critical care echocardiography [27]. We perform TEE examination in all ventilated patients with septic shock and no contraindication for esophageal intubation, in conjunction with TTE for tissue Doppler assessment as a standard of care. All measurements were performed on a dedicated workstation (Xcelera™, Philips) by an independent expert who was unaware of patients’ data and outcome.

In the four-chamber view, the following echocardiographic parameters were measured: LV ejection fraction (EF) calculated conventionally from both the LV end-diastolic volume (LVEDV) and LV end-systolic volume (LVESV) using the monoplane modified Simpson’s rule [28]; maximal velocity of mitral Doppler *E* and *A* waves recorded at the tip of valvular leaflets, and *E*/*A* ratio (sinus rhythm); tissue Doppler imaging *E*′ wave maximal velocity measured on the lateral mitral annulus, and lateral *E*/*E*′ ratio [29]; right ventricular (RV) and LV end-diastolic area ratio (RVEDA/LVEDA) [30]. Pulse wave Doppler velocity–time integral of LV outflow tract (LVOT VTI) was measured in the apical five-chamber view or in the 120° transgastric view to indirectly assess LV stroke volume. The collapsibility index of the superior vena cava (ΔSVC) was measured using TEE M-mode in the long-axis view of the great vessels to assess fluid responsiveness [31]. A potential paradoxical motion of the interventricular septum was identified in the short-axis view of the heart. Its association with RV dilatation (i.e., RVEDA/LVEDA > 0.6) defined acute cor pulmonale [30].

LV diastolic dysfunction was defined by the presence of a lateral *E*′ maximal velocity <10 cm/s [32]. LV dilatation was defined by an indexed LV end-diastolic volume (LVEDVi) >75 ml/m^2^, irrespective of gender [28].

### Statistical analysis

Results are presented as means ± standard deviations and percentages. Quantitative variables were compared between patients who died in the ICU and patients who were discharged alive using Student’s *t* test or Mann and Whitney’s test when data were not normally distributed. Qualitative variables were compared between groups using the *χ*^2^ test or Fisher’s exact test, when necessary.

A stepwise multivariate logistic regression analysis was performed to identify independent variables associated with ICU mortality. All variables with a *p* value <0.20 in the univariate analysis were incorporated into the model. The significance of parameters which remained in the final model was tested after adjusting for the SAPS II score. Odds ratio and their 95 % confidence intervals (CI) were determined. To assess the potential impact of the severity of LV diastolic dysfunction on prognosis, patients were divided based on quartiles of lateral *E*′ maximal velocity. A two-tailed *p* value <0.05 was considered statistically significant.

## Results

Five hundred and forty patients were admitted to our ICU with a septic shock during the study period. In 304 of them (56 %), echocardiography was not available due to the absence of digital recordings or to a low image quality precluding accurate measurements, and 12 patients (2 %) failed to meet all diagnostic criteria of septic shock. Finally, 223 patients were included into the study (140 men [63 %]; age 64 ± 13 years; SAPS II 55 ± 18; SOFA 10 ± 3; Charlson 3.5 ± 2.5) and 204 of them (91 %) were under invasive mechanical ventilation. The source of infection was documented in 185 patients (83 %), and a bacteremia was identified in 85 patients (38 %). Sepsis originated from intra-abdominal infection in 87 patients (39 %), a pulmonary infection in 48 patients (22 %), an urogenital infection in 37 patients (17 %) and a skin or joint and bones infection in 12 patients (5 %). Seventy-eight patients died in the ICU, accounting for a 35 % mortality rate.

When compared with survivors, patients who died in the ICU had significantly higher SAPS II and SOFA scores and had more frequently an underlying hematologic malignancy (Table 1). No significant inter-group difference in infection characteristics was found. Patients who died in the ICU had significantly lower pH (7.27 ± 0.17 vs. 7.32 ± 0.12: *p* = 0.03), higher lactate (6.3 ± 5.3 vs. 4.1 ± 3.7 mmol/L: *p* = 0.0004) and creatinine levels (225 ± 144 vs. 180 ± 140 µmol/L: *p* = 0.03) and exhibited more frequently a disseminated intravascular coagulation (10/78 [13 %] vs. 11/145 [8 %]: *p* = 0.002) at the time of shock diagnosis than their counterparts (Table 1). The delay of initial antibiotic therapy was longer (4.3 ± 10.6 vs. 1.8 ± 4.0 h: *p* = 0.02), and antibiotics were less frequently appropriate (63/78 [81 %] vs. 135/145 [93 %]: *p* = 0.005) in non-survivors than in patients discharged alive from the ICU. Similarly, patients who died in the ICU received a greater dose of vasopressors (1.25 ± 1.17 vs. 0.55 ± 0.47 µg/kg/min: *p* < 0.0001) than survivors and required more frequently the administration of epinephrine (35/78 [45 %] vs. 33/145 [23 %]: *p* = 0.0006), renal replacement therapy (19/78 [24 %] vs. 19/145 [13 %]: *p* = 0.04) and mechanical ventilation (77/78 [99 %] vs. 127/145 [88 %]: *p* = 0.0012) (Table 1). After initial fluid resuscitation, hemodynamic parameters were not significantly different between groups (Table 2). While mean LVEF, mean LVEDV and mean ΔSVC were similar between groups, non-survivors exhibited slightly yet significantly lower LV stroke volume reflected by a decreased mean LVOT VTI (16 ± 6 vs. 17 ± 5 cm: *p* = 0.03) and larger RV cavity, as reflected by a significantly greater RVEDA/LVEDA ratio (0.77 ± 0.83 vs. 0.59 ± 0.17: *p* = 0.01) without associated paradoxical septal motion (Table 2). The prevalence of hyperkinetic LV (LVEF > 70 %) was low in the two study groups (2/78 [3 %] vs. 10/145 [7 %]: *p* = 0.17). Actual LV dilatation was rarely observed, with the same frequency in both groups (8/78 [10 %] vs. 14/145 [10 %]: *p* = 0.89). Mean value of lateral *E*′ maximal velocity was significantly lower in patients who died in the ICU when compared with survivors (10.5 ± 3.7 vs. 11.9 ± 4.5 cm/s: *p* = 0.04), whereas mean *E*/*E*′ lateral only tended to be higher in those patients without reaching statistical significance (Table 2). The proportion of LV diastolic dysfunction tended to be higher in non-survivors than in their counterparts, even if the difference was not significant (28/78 [36 %] vs. 41/145 [28 %]: *p* = 0.15). In the lower quartile of *E*′ lateral maximal velocity values (*E*′ lateral ≤ 8.2 cm/s), the proportion of deceased patients tended to be higher, whereas the opposite trend was observed in the higher quartile (*E*′ lateral ≥ 14.1 cm/s) (Table 3).

**Table 1:** Clinical characteristics of the study population

Parameters	Global population (n = 223)	Survivors (n = 145)	Non-survivors (n = 78)	*p*
*Patients characteristics*				
Age (years)	64 ± 13	63 ± 13	64 ± 13	0.74
Male (n)	140 (63)	93 (64)	47 (60)	0.57
SAPS II	55 ± 18	49 ± 15	66 ± 17	<0.0001
SOFA	10 ± 3	9 ± 3	11 ± 3	<0.0001
Charlson	3.5 ± 2.5	3.4 ± 2.6	3.6 ± 2.1	0.56
Cancer (n)	35 (16)	23 (16)	12 (15)	0.93
Hematologic malignancy (n)	10 (4)	1 (0.7)	9 (12)	0.0004
Chronic renal failure (n)	14 (6)	7 (5)	7 (9)	0.22
Cirrhosis (n)	12 (5)	5 (3)	7 (9)	0.08
*Infection characteristics*				
Documented infection	185 (83)	123 (85)	62 (80)	0.31
Bacteremia	105 (47)	65 (45)	40 (51)	0.36
Abdominal	87 (39)	57 (41)	30 (41)	0.96
Pulmonary	48 (22)	31 (22)	17 (23)	0.94
Urogenital	37 (17)	26 (19)	11 (15)	0.46
Skin or joint and bones	12 (5)	1 (7)	3 (4)	0.14
Other origin	30 (13)	16 (13)	13 (15)	0.21
*Biology*				
pH	7.30 ± 0.14	7.32 ± 0.12	7.27 ± 0.17	0.03
Lactates (mmol/L)	4.9 ± 4.4	4.1 ± 3.7	6.3 ± 5.3	0.0004
PaO_2_/FiO_2_	177 ± 113	180 ± 116	171 ± 108	0.57
Creatinine (µmol/L)	196 ± 143	180 ± 140	225 ± 144	0.03
Bilirubin (µmol/L)	22 ± 35	20 ± 24	27 ± 49	0.12
ALAT (IU/L)	134 ± 335	115 ± 302	167 ± 389	0.28
Hemoglobin (g/dL)	11 ± 2	11 ± 2	11 ± 3	0.69
White blood cells (G/L)	15.5 ± 14.5	15.5 ± 12.3	15.6 ± 18.1	0.93
Platelets (G/L)	211 ± 151	217 ± 157	199 ± 138	0.40
DIC (n)	21 (9)	11 (8)	10 (13)	0.002
*Treatment characteristics*				
Delay of antibiotic therapy (h)	2.7 ± 7.1	1.8 ± 4.0	4.3 ± 10.6	0.02
Antibiotics administered <3 h	174 (78)	119 (84)	55 (71)	0.06
Appropriate initial antibiotic therapy	198 (88)	135 (93)	63 (81)	0.005
Fluid therapy (L)	4.4 ± 3.7	4.4 ± 3.6	4.6 ± 3.9	0.74
Noradrenaline (n)	205 (92)	134 (95)	71 (91)	0.72
Epinephrine (n)	68 (30)	33 (23)	35 (45)	0.0006
Dobutamine (n)	11 (5)	6 (4)	5 (6)	0.45
Maximal dose of vasopressors^a^ (µg/kg/min)	0.79 ± 0.85	0.55 ± 0.47	1.25 ± 1.17	<0.0001
Hydrocortisone Hemisuccinate (n)	22 (10)	11 (8)	11 (14)	0.12
Renal replacement therapy	38 (17)	19 (13)	19 (24)	0.04
Invasive mechanical ventilation	204 (91)	127 (88)	77 (99)	0.0012

Results are expressed as means ± standard deviations; numbers in brackets are percentages

*SAPS II* Simplified Acute Physiology Score, *SOFA* Sequential Organ Failure Assessment, *ALAT* alanine aminotransferase, *DIC* disseminated intravascular coagulation

^a^During the ICU stay

**Table 2:** Hemodynamic parameters and echocardiographic findings at the time of assessment

Parameters	Global population (n = 223)	Survivors (n = 145)	Non-survivors (n = 78)	*p*
*Hemodynamic parameters*				
sBP (mmHg)	112 ± 29	113 ± 28	109 ± 31	0.34
dBP (mmHg)	63 ± 17	64 ± 16	62 ± 18	0.36
mBP (mmHg)	77 ± 20	78 ± 19	75 ± 22	0.35
HR (bpm)	109 ± 25	107 ± 24	112 ± 27	0.19
CVP (mmHg)	11 ± 5	11 ± 5	11 ± 6	0.72
*Echocardiography*				
LVEF (%)	48 ± 17	49 ± 17	46 ± 18	0.39
LVEDV (mL)	93 ± 36	95 ± 37	90 ± 34	0.37
LVOT VTI (cm)	16 ± 5	17 ± 5	16 ± 6	0.03
*E* wave (cm/s)	80 ± 27	80 ± 25	79 ± 30	0.49
*A* wave (cm/s)	82 ± 27	82 ± 27	82 ± 27	0.89
*E*/*A*	1.03 ± 0.55	1.05 ± 0.58	0.99 ± 0.46	0.85
Lateral *E*′ wave (cm/s)	11.4 ± 4.3	11.9 ± 4.5	10.5 ± 3.7	0.04
*E*/*E*′ lateral	8.0 ± 3.8	7.7 ± 3.7	8.5 ± 4.0	0.26
RVEDA/LVEDA	0.66 ± 0.51	0.59 ± 0.17	0.77 ± 0.83	0.01
Paradoxical septal motion (n)	14 (6)	7 (5)	7 (9)	0.23
ΔSVC (%)	14 ± 13	15 ± 13	14 ± 12	0.97

Results are expressed as means ± standard deviations; numbers in brackets are percentages

*sBP* systolic blood pressure, *mBP* mean blood pressure, *dBP* diastolic blood pressure, *HR* heart rate, *CVP* central venous pressure, *LVEF* left ventricular ejection fraction, *LVEDV* left ventricular end-diastolic volume, *LVOT VTI* left ventricular outflow tract velocity–time integral, *RVEDA* right ventricular end-diastolic area, *LVEDA* left ventricular end-diastolic area, *ΔSVC* collapsibility index of the superior vena cava

**Table 3:** Distribution of patients according to quartiles of lateral *E*′ maximal velocity

Quartiles	Survivors (n = 145)	Non-survivors (n = 78)	*p*
Lateral *E*′ ≥ 14.1 cm/s	31 (21)	11 (14)	0.14
10.9 ≥ lateral *E*′ < 14.1 cm/s	30 (21)	13 (17)	
8.2 ≥ lateral *E*′ < 10.9 cm/s	17 (12)	13 (17)	
8.2 ≥ lateral *E*′ < 14.1 cm/s	21 (14)	20 (26)	

Numbers in brackets are percentages

In the multivariate analysis, the inappropriateness of initial antibiotic therapy, the maximal dose of vasopressors during ICU stay, the SOFA score and *E*′ lateral maximal velocity (OR 1.12 [95 % CI 1.01–1.24] per decrease of 1 cm/s; *p* = 0.02) were significantly associated with ICU mortality (Table 4). After adjusting for the SAPS II score, the initial inappropriate antibiotic therapy and the maximal dose of vasopressors remained independent factors related to ICU mortality. In contrast, a trend was only observed for lateral *E*′ maximal velocity (OR 1.11 [CI 95 % 0.99–1.23]: *p* = 0.07) (Table 4).

**Table 4:** Multivariate analysis to determine prognostic factors associated with ICU mortality

Parameters	Odds ratio [95 % CI]	*p*
*Without adjustment*		
Inappropriate initial antibiotic therapy	4.17 [1.33–12.5]	0.03
Maximal dose of vasopressors (per mg/h)	1.38 [1.16–1.63]	0.01
SOFA (per point)	1.16 [1.02–1.32]	0.02
Lateral *E*′ maximal velocity (per decrease of 1 cm/s)	1.12 [1.01–1.24]	0.02
*When adjusting for SAPS II*		
Inappropriate initial antibiotic therapy	5.88 [1.64–20.00]	0.007
Maximal dose of vasopressors (per mg/h)	1.26 [1.08–1.47]	0.004
Lateral *E*′ maximal velocity (per decrease of 1 cm/s)	1.11 [0.99–1.23]	0.07

*SOFA* Sequential Organ Failure Assessment, *SAPS* Simplified Acute Physiology Score

## Discussion

In the present study, independent factors related to ICU mortality in septic shock patients were the inappropriate initial antibiotic therapy, the maximal dose of vasopressors used during ICU stay, the SOFA score and a decreased lateral *E*′ maximal velocity indicating altered LV diastolic properties. After adjusting for the SAPS II score, inappropriate antibiotic therapy and maximal dose of vasopressors were the sole factors independently associated with ICU mortality, while a trend was only observed for lateral *E*′ maximal velocity.

LV diastolic dysfunction was observed in 31 % of our patients, in keeping with the prevalence reported previously in patients hemodynamically assessed for severe sepsis and septic shock, which ranged between 20 % [15, 16] and 57 % [33]. Such variations may be related to the use of different definitions for LV diastolic dysfunction, different timing of echocardiographic assessment relative to the sepsis course, and distinct clinical settings (i.e., severe sepsis vs. septic shock). Using the currently recommended criteria [32], we and others previously identified a reversible LV diastolic dysfunction in 20 % of septic shock patients [15, 16]. The marked smaller sample size of these previous studies when compared with the present cohort may account for this discrepancy. The current study confirms that true LV enlargement is uncommon in septic shock patients [15, 34]. The potential protective effect of LV cavity dilatation on outcome has long been suggested in septic patients [7]. Its prognostic significance is currently debated and remains to confirm in large-scale studies [35].

Measurement of tissue Doppler *E*′ wave maximal velocity at the mitral annulus reflects the lengthening velocity of LV longitudinal myocardial fibers during early diastole. Accordingly, a reduced *E*′ velocity <8 cm/s when measured on septal mitral annulus and <10 cm/s when measured on lateral mitral annulus are diagnostic of LV diastolic dysfunction [32]. We previously showed the high precision of this measurement in critically ill patients, as reflected by a 4 and 2 % inter- and intra-observer reproducibility, respectively [29]. Accordingly, the small absolute difference in lateral *E*′ maximal velocities observed between groups in the current study is greater than expected reproducibility of measurement. We purposely measured *E*′ maximal velocity on the lateral aspect of the mitral annulus because we previously found that this parameter was relatively independent of LV loading conditions, as opposed to septal *E*′ maximal velocity [29]. Nevertheless, Mahjoub et al. [36] recently reported that the increase in *E*′ maximal velocity secondary to fluid loading was significantly greater in septic patients with fluid responsiveness, whereas non-responders to a fluid challenge exhibited larger increase in *E*/*E*′ ratio reflecting higher LV filling pressure after blood volume expansion. In our patients, mean ΔSVC, which has previously been validated as a marker of fluid responsiveness [31], was similar in survivors and in non-survivors. Moreover, mean fluid volume administered to our septic patients prior to hemodynamic assessment was also similar in both groups. Accordingly, fluid resuscitation has presumably not influenced lateral *E*′ maximal velocity in the present cohort of septic shock patients.

Although LV diastolic dysfunction alters the prognosis of many cardiac diseases [12, 13], its prognostic impact in septic shock remains controversial. Several studies failed to identify LV diastolic dysfunction as a potential predictor of outcome in septic patients [10, 15]. In contrast, a recent meta-analysis reported a relationship between abnormal LV relaxation as assessed by *E*′ maximal velocity and death in septic patients [17]. In a cohort of 262 patients with severe sepsis and septic shock, Landesberg et al. [9] showed that LV diastolic dysfunction was the strongest predictor of short-term mortality, even after adjusting for the APACHE II score. ICU mortality was also related to the severity of LV diastolic dysfunction [9]. In keeping with these results, lateral *E*′ maximal velocity was independently associated with ICU mortality in our septic shock patients. Nevertheless, a nonsignificant trend only persisted when adjusting for the SAPS II score and was observed when dividing the current cohort according to quartiles of lateral *E*′ maximal velocity. A similar trend was observed when using a cutoff value of 10 cm/s for defining LV diastolic dysfunction [32] and when comparing its prevalence between non-survivors and survivors. The potential impact of LV diastolic dysfunction on ICU mortality of septic shock patients may be related to various factors. Abnormal relaxation results in a reduced early LV filling, an increased contribution of left atrial contraction to late LV filling and a progressive increase in LV filling pressure [14]. Marked hypovolemia, tachycardia and high rate of acute atrial fibrillation in sepsis [37] contribute to further reduce early LV filling by shortening the duration of diastole and subsequently to decrease cardiac output. The relative increase in LV filling pressures associated with diastolic dysfunction could facilitate a positive fluid balance, which prognostic impact has recently been suggested [38, 39]. This may worsen non-cardiogenic pulmonary edema associated with sepsis [40], or even lead to right cardiac failure [18], as suggested by the increased RVEDA/LVEDA ratio without associated acute cor pulmonale in our patients who died in the ICU.

As previously reported [26], inappropriate initial antibiotic therapy was the strongest predictor of ICU mortality in the present cohort of septic shock patients. In our patients, the 4.17 odds ratio for ICU death was in agreement with the fivefold higher risk of death observed by Kumar et al. [26]. Inappropriate initial antibiotic therapy remained significantly associated with ICU mortality after adjusting for SAPS II score with a 5.88 odds ratio. In addition to the SOFA score, the maximal dose of vasopressors administered to our septic shock patients was also an independent risk factor for ICU mortality. Higher mean dose of vasopressors administered to non-survivors may reflect the presence of a more severe circulatory failure with profound vasoplegia, which has long been related to unfavorable outcome [7]. Higher mortality rate in septic shock patients presenting with a hyperkinetic hemodynamic profile associating markedly increased cardiac output and low systemic vascular resistance has also been reported in echocardiographic studies [34, 41]. In our patients, mean LVEF was similar between survivors and non-survivors, and the prevalence of hyperkinetic profile was not significantly different between groups. The low prevalence of marked hyperkinetic states reflected by a LVEF > 70 % in septic shock patients presumably explains this result due to our underpowered study [34]. Nevertheless, currently available studies have been conducted on limited sample size [7, 34], or failed to enroll consecutive patients [41]. Accordingly, the prognostic value of LV hyperkinesia as a marker of profound sepsis-induced vasoplegia remains to confirm in a large prospective cohort of consecutive patients with septic shock who are hemodynamically assessed using echocardiography.

Although various other factors have been shown to be prognostic in patients with septic shock, including comorbidities [42], site of infection [43], bacteriological documentation [44], causative microorganism [45] and initial management [25], none of these factors was associated with ICU mortality in the present cohort of septic shock patients.

The present study has several limitations. Due to its retrospective design, the absence of available echocardiographic recordings in a substantial proportion of eligible patients constitutes a selection bias. Nevertheless, our cohort appears representative of septic shock patients admitted to the ICU, with a 35 % mortality rate [1, 2], a large proportion of patients under ventilator [43, 44] and a mean SOFA score of 10 reflecting the presence of sepsis-induced multiple organ failure [44]. Since LV diastolic dysfunction is commonly associated with age, high blood pressure, diabetes and ischemic heart disease, it may be present prior to the development of septic shock in a large proportion of patients, rather that induced by sepsis [14]. The potential worsening of LV diastolic function secondary to the development of septic shock and its reversibility with the control of the causative infection could not be documented in the present study since echocardiography was not serially performed. Nevertheless, no prospective study has yet repeatedly assessed LV diastolic properties in a large cohort of septic shock patients. Accordingly, only few information on the course of LV diastolic properties during the management of septic shock is currently available, and the reversibility of sepsis-induced LV diastolic dysfunction remains to be confirmed [15, 16]. Since septal *E*′ maximal velocity was not measured in our patients, we could not use the traditional diagnostic criteria of LV diastolic dysfunction based on mean septal and lateral *E*′ maximal velocities and mean *E*/*E*′ ratio [32]. Nevertheless, *E*′ maximal velocity appears to be less dependent of LV loading conditions when recorded at the lateral rather than at the septal mitral annulus [29]. Finally, the dose of vasopressors and inotropes which may have altered LV diastolic properties at the time of echocardiographic assessment has not been recorded.

## Conclusion

The present study confirms the frequency of LV diastolic dysfunction at the initial phase of septic shock. LV diastolic function assessed by the measurement of lateral *E*′ maximal velocity was independently associated with ICU mortality, in conjunction with the inappropriateness of the initial antibiotic therapy, the maximal dose of vasopressors administered and the SOFA score. After adjusting for the SAPS II score, a nonsignificant trend was only observed for *E*′ lateral maximal velocity. Due to the retrospective design of the present study and limitations of currently reported series, a prospective multicenter study allowing the longitudinal assessment of a large cohort of patients with septic shock using serial echocardiographic examinations is required to definitely confirm the potential prognostic role of LV diastolic dysfunction in this highly prevalent disease.

## Key messages

- Left ventricular diastolic dysfunction is frequently observed in ICU patients who are hemodynamically assessed using echocardiography for a septic shock.
- Left ventricular diastolic function assessed using lateral *E*′ maximal velocity was independently associated with ICU death in conjunction with inappropriate initial antibiotic therapy, maximal dose of vasopressors and SOFA score.
- After adjusting for SAPS II score, only a nonsignificant trend was observed between lateral *E*′ maximal velocity and ICU mortality.
- A large-scale multicenter study is needed to definitely confirm the prognostic role of left ventricular diastolic dysfunction in septic shock patients.
